# Revisiting the Walburg hypothesis that radiation affects only tumor deaths but not non-tumor deaths in mice

**DOI:** 10.1093/jrr/rrag063

**Published:** 2026-09-16

**Authors:** Nori Nakamura, Satoshi Tanaka, Ignacia Braga Tanaka, Yoshiya Shimada

**Affiliations:** Department of Molecular Biosciences, Radiation Effects Research Foundation, Hiroshima, Japan; Department of Radiobiology, AMBIC, Institute for Environmental Sciences, Rokkasho, Aomori, Japan; Department of Radiobiology, AMBIC, Institute for Environmental Sciences, Rokkasho, Aomori, Japan; Department of Radiobiology, AMBIC, Institute for Environmental Sciences, Rokkasho, Aomori, Japan

**Keywords:** radiation, carcinogenic effect, mutation hypothesis, early onset hypothesis

## Abstract

Starting in the 1950s or earlier, irradiation was found to shorten lifespans in mice, which raised the possibility that radiation science might contribute to an understanding of the nature of aging. However, in 1975, Walburg published a review paper to express his hypothesis that radiation exposure increases the risk of tumor deaths but not for non-tumor deaths. Subsequently, it became a mainstream belief that irradiation induces oncogenic mutations and thereby increases the risk of cancer and thus life shortening. However, we now think that the Walburg’s hypothesis is not tenable as it did not include contradictory studies that were available before 1975, and additional contradictory papers were published after 1975; namely, all irradiated individuals are affected. If we accept the notion that irradiation shortens the lifespan of all individuals regardless of the cause of death, it suggests that irradiation may accelerate general aging. Based on these considerations and other available evidence in the field, the mutation induction theory appears untenable as the sole mechanism in radiation carcinogenesis. Under such conditions, the old hypothesis that radiation induces accelerated aging should be revived. Current knowledge in the field of natural aging supports the hypothesis that radiation causes DNA breaks, most of which are correctly repaired, but not at the chromatin level (chromatin fatigue) which leads to accelerated aging. The critical difference from the traditional mutagenesis model is that no stem cell mutagenesis is necessary to consider.

## INTRODUCTION

Early studies on rodents on the somatic effects of radiation found an increase in mortality and a consequent shortening of the mean lifespan, which appears to be an acceleration of natural aging [1, 2]. Natural aging is a biological process that is difficult to understand, and thus if irradiation shortens the lifespan, it was once felt that radiation science might contribute to an understanding of the aging process. (It is mentioned that the following arguments are not concerned with the effect of high doses of radiation which may cause acute deaths within 30 days after the exposure.) In those days, however, not enough information was available to determine whether the observed increase in mortality was attributable to the induction of malignancies or to accelerated aging (i.e. an earlier onset of diseases). In 1975, Walburg published a review paper indicating that the effect of irradiation is restricted to tumor deaths and not to non-tumor deaths: that is, non-specific aging does not occur [3]. Following the discoveries of oncogenes and tumor suppressor genes in the 1980s, the induction theory of malignancy became a mainstream in the field of radiation carcinogenesis [4, 5].

When Walburg drafted his review paper, however, clean breeding conditions for mice (specific-pathogen-free conditions) were not yet established and the strains of mice used were limited which proved to be well responsive to carcinogen treatments (e.g. RFM or LAF1 mice). Also, the review failed to include some mouse data that were available in 1975. Nevertheless, this Walburg hypothesis was the beginning of the mainstream hypothesis concerning the mechanisms involved in radiation carcinogenesis that lasted for many years. However, there are new results that are incompatible with that hypothesis, and this includes epidemiologic studies of atomic-bomb survivors which started to show a significant increase in non-cancer risks. Therefore, it was felt important to revisit the Walburg paper in the light of more recent findings.

The paper was published in 1975 and was titled ‘Radiation-induced life-shortening and premature aging,’ and suggested that after adjusting for the competing probabilities of death, radiation exposure affects tumor deaths but not non-tumor deaths [3]. In other words, radiation does not cause an acceleration of non-specific aging. In this regard, the UNSCEAR 1977 Report said: ‘…*that the concept of a non-specific ageing effect of radiation is no longer tenable. At present, the consensus seems to be that life-span shortening is to be attributed almost entirely to the induction of neoplasia, especially at low doses and dose rates*’ [4]. Later, the UNSCEAR 1982 Report (section 58) said: ‘*The older theories were discussed by Walburg and their applicability to a possible effect of premature aging was criticized as mortality data and causes of death in irradiated animals indicate a life-shortening action essentially related to tumor induction*’ [5].

Also, the UNSCEAR 1982 Report (section 41) said that ‘*Though never defined clearly, the words specific and non-specific (in terms of aging) have therefore been taken to indicate neoplastic and non-neoplastic contributions to life shortening*’ [5]. Thus, if it could be found that irradiation shortens the lifespan of mice dying from non-tumor causes as well, it implies that irradiation caused non-specific aging, and this forces us to find new explanations other than the mutagenesis model which was assumed to be the mechanism of radiation carcinogenesis.

Here are three reasons why we think Walburg’s conclusion is untenable.

First, mouse survival curves most often shift in a parallel manner toward younger ages following radiation exposures e.g. [6–10]. If an exposure to radiation affects only a fraction of the irradiated animals as the mutagenesis model assumes, and the proportion of the affected individuals increases with an increase in radiation dose, then the survival curves would not shift in a parallel manner from the control non-irradiated curves but must show gradually declining slopes, which is not the case.

Second, no report ever showed a dose related increase in the fraction of mice that underwent tumor death. In this context, there are reports describing dose-related increases in the proportion of tumor deaths in mice. However, such reports showed either prematurely terminated results e.g. [11] or corrected data to adjust for the shortened lifespan of the irradiated group e.g. [12], and both treatments led to a misinterpretation of the results [13].

Third, at least four studies showed radiation-induced life shortening occurs in mice dying from both tumor and non-tumor deaths [8, 14–16].

We now wish to discuss individual details concerning these observations.

## RESULTS

### A brief history of the relationship between natural aging and radiation-related aging (page 149 of the Walburg paper)

A brief summary of this section is shown below. Although there is nothing to argue about, one thing is notable: namely, there were reports before 1975 which indicated that all irradiated mice underwent life shortening.

The first observation that radiation-exposed rats showed increased mortality rates and looked old was made in 1939. Subsequently, in 1957 Alexander [1] and Upton [2] developed the idea that this life shortening effect of radiation may be related to natural aging. At that time, it was recognized that irradiation can (1) increase mortality, and all diseases appeared to develop earlier, (2) accelerate external characteristics of aging, (3) cause early onset of cataracts and (4) decrease fertility, etc.

In the 1960s, two large-scale studies attracted interest, and the above-mentioned observations were largely reproduced. The important point was that radiation-related life shortening is not caused by the induction of some specific diseases, but from all causes of death (namely, the acceleration of natural aging). For example, Alexander [1] observed life shortening effects of radiation, even among tumor-free individuals in LAF1 mice exposed to 400 R of X rays. Also, Upton et al. noted that, other than thymic lymphoma, the onset age for all types of malignancy advanced similarly, and incidence rates of all malignancies did not increase linearly with the dose [17].

Furthermore, Lindop and Rotblat suggested that, after careful necropsy of the animals, all causes of death were advanced in time [18]. However, because the relative ages at the onset of different diseases were not the same as those tumors induced in the irradiated population (such as thymic lymphoma or leukemia), they carefully concluded that radiation aging cannot be identical to natural aging.

### Understanding the cause of death is indispensable (page 153 of the Walburg paper)

A summary of this section is as follows:

In the 1960s, there were publications which indicated the presence of radiation-induced non-specific aging based solely on the increased mortality rate following irradiation but without pathologic data. Walburg claimed that such data cannot be the proof for accelerated aging. This is because among the mouse strains used, some expressed a high sensitivity to radiation-induced thymic lymphoma, leukemia or ovarian tumors in their early life which could lead to a decrease in the number of mice dying from other tumors which occur later in life. Thus, it is important not to argue about the acceleration of non-specific aging just from the survival curves alone, and pathology data is needed. This complaint by Walburg is fully accepted.

### Pathology data is powerful for tumor associated deaths, but not for non-tumor deaths (page 155 of the Walburg paper)

If a large tumor was found at autopsy, it is relatively easy to determine the possible cause of death. However, if tumors were either small or not detected, then, it would be difficult to find the cause of death. Walburg wrote that ‘*few data exist assessing the effect of radiation on specific nonneoplastic diseases*’ (page 156), which also applies today. It is also mentioned that in epidemiologic studies in humans, increased risks for cancer were observed among radiologists and among atomic-bomb survivors but not for non-cancer deaths. However, we now know that non-cancer risks are also significantly increased among the survivors [19].

### Adjustment for competing risks: Mean age at death may or may not be artifactual (page 156 of the Walburg paper)

Here, Walburg complained about the lack of adjustments for competing probabilities of death in early studies. Certainly, this makes sense because some strains of mice develop thymic lymphoma or leukemia etc. in early life following a radiation exposure. However, we now know that depending on the strain of mice and modality of radiation exposure (low dose or low dose-rate exposure), one can optimize exposure conditions so that the proportions of the causes of death will not change as a result. In this situation, adjustments would not be necessary because the competing probabilities of death must act equally on both the control and exposed groups such that a shortened life span would be the only appropriate expression of radiation effect. For example, experimental results by Tanaka *et al.* [8] and Store [20] showed no alterations in the proportions of the causes of death following radiation exposures. Tanaka *et al.* [8] wrote that ‘*Since there was almost no increase in the incidence of most neoplasms that caused death in individual mice, the life shortening observed … was due to the early development of these neoplasms*’ and ‘*not from increased incidence of specific neoplasms*’.

It is noted that this adjustment of competing risks is only performed in rodent experiments and is not usually applied in human epidemiology, and this is related to the fact that certain strains of mice (e.g. RFM or LAF1 mice) develop specific malignancies such as thymic lymphoma, etc. at high frequencies after an exposure to radiation [21]. To avoid such problems, researchers started to use hybrid mice from two inbred strains such as B6C3F1 mice, apply ˂2 Gy of radiation to avoid the development of thymic lymphoma [22], or expose animals to low dose-rate radiation [7]. These considerations decreased the confusion associated with interpretation of the results.

### All diseases advance in time or not by radiation (page 158 of the Walburg paper)

Walburg cited an example that radiation effects disappeared when thymic lymphosarcoma (TLS) and myeloid leukemia (ML) were excluded from the data; namely, the mortality curve without TLS and ML overlapped or nearly coincided with the control curve. (Fig. 1 is a reproduction of Fig. 2 of the Walburg paper). However, caution must be exercised in interpreting these data since the mice used in this experiment were RFMf/Wg males that were known to be highly sensitive to radiation induction of ML (1% in the control group but up to 40% after a 2–3 Gy exposure) [23–25]. Therefore, the results cannot be regarded as representing the general response of mice following an exposure to radiation. In this regard, Walburg described that ‘*more documentation and larger sample size in different strains of mice … are necessary for confirmation*’, and we now know the answers.

**Fig. 1:**
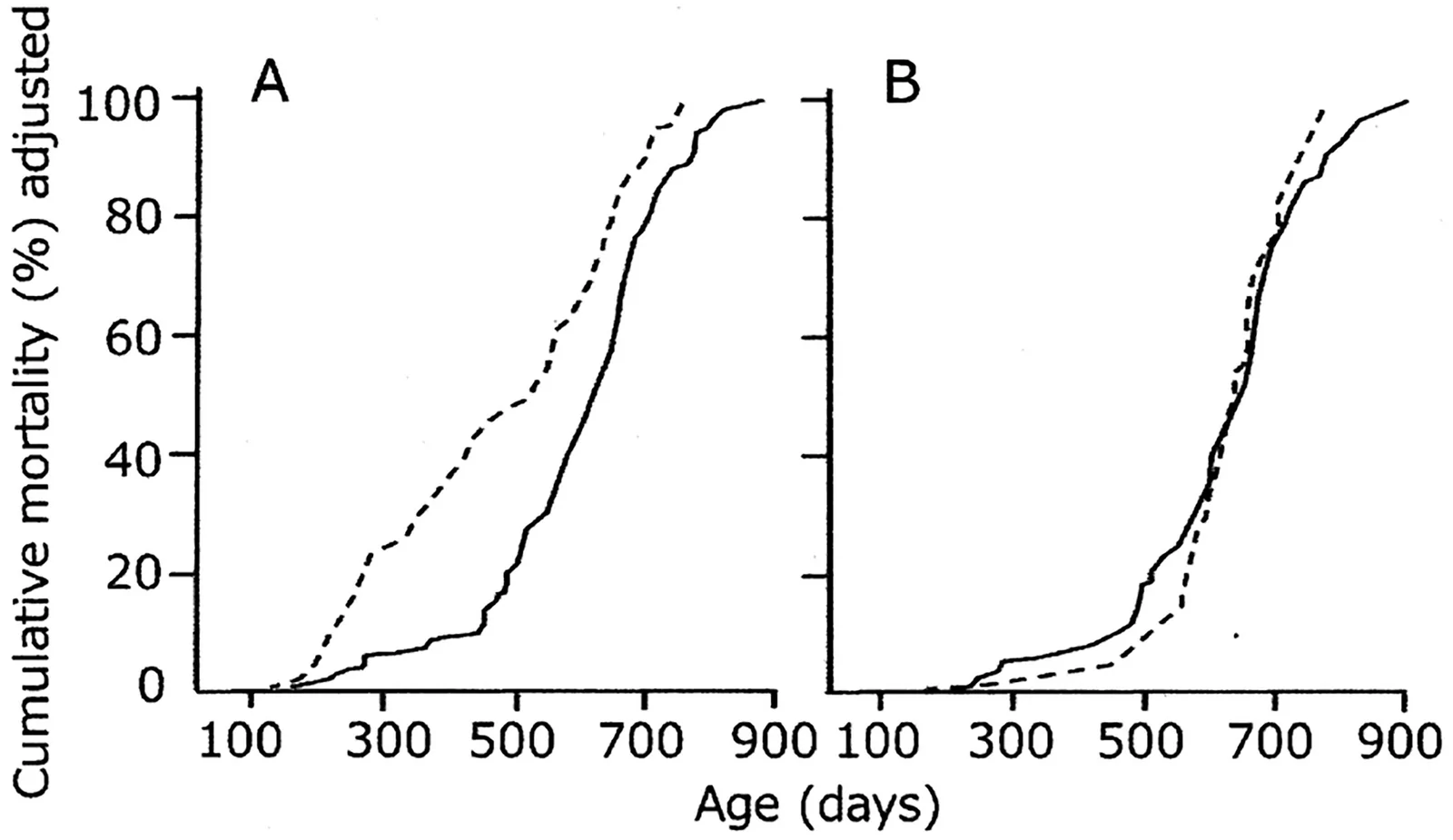
Cumulative mortality (%) for RFMf/Wg male mice exposed to 300 R of X rays and raised in a conventional environment. The Y-axis represents adjusted mortality using the Kaplan–Meier method. (A) Represents all deaths, (B) represents all deaths except thymic lymphoma and myeloid leukemia. Solid lines represent control groups, and broken lines represent exposed group. (Fig. 2 of the Walburg paper [3]).

**Fig. 2:**
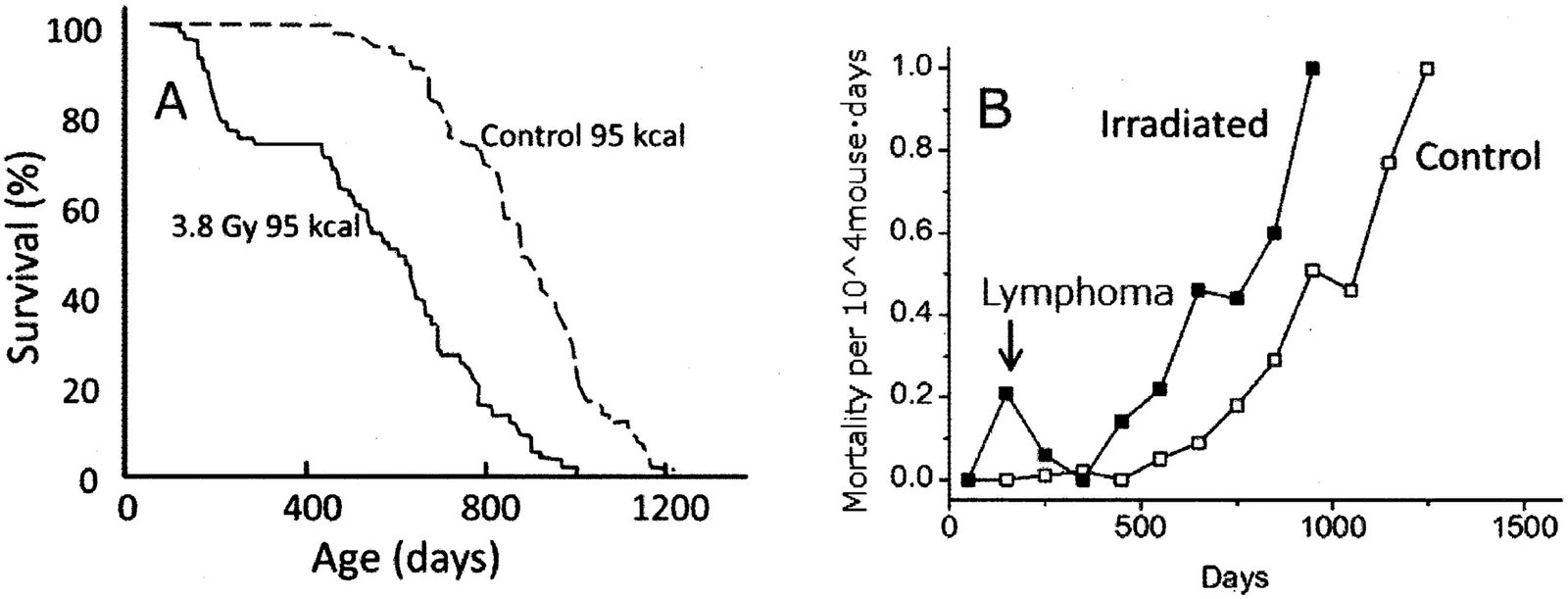
(A) Survival curve for B6C3F1 male mice exposed to 3.8 Gy of X rays. Solid line indicates the exposed group, and the broken line indicates the controls [26]. (B) Mortality rate calculated from the survival curves in A.

Here are at least three studies which contradict the notion that no radiation effect remains if hematologic diseases were excluded.

(1) Storer irradiated female BDF1 mice with 0, 100, 300 and 500 R of X rays, and raised the animals until ˃80% of them died a natural death [20]. The results did not show alterations in the distributions of the cause of death (leukemia, other tumors and non-tumors) in response to the dose (Table 1), but the mean lifespan was shortened in proportion to the dose. This study was published in 1965, 10 years prior to the Walburg paper, in Radiation Research but was not mentioned in the Walburg paper.

**Table 1:** Incidence of leukemia and other tumors in samples of mice autopsied [20]

Group	No. of mice autopsied	Leukemia^a^ (%)	Other tumors^b^ (%)	Non-tumorous (%)
Control	74	26	28	46
100 R	115	26	40	34
300 R	104	27	31	42
500 R	118	36	22	42

This table shows that radiation exposure did not increase the proportion of mice dying from leukemia or other tumors.

^a^Primarily thymic lymphoma.

^b^Principally ovarian and mammary tumors.

(2) Tanaka *et al.* reported on their large-scale study of chronic long-term exposures of B6C3F1 mice [8]. The study consisted of three different dose-rate groups plus a control group. Irradiation lasted for 400 days, and each group consisted of 500 males and 500 females. The highest dose-rate group (exposed to an average of 20 mGy/day to a total dose of 8 Gy) showed ~100 days of life shortening, with no change in the spectrum for the causes of death. An interesting point is that ~30% of B6C3F1 mice develop malignant lymphoma in both the radiation exposed and control groups, but exclusion of lymphoma does not lead to the disappearance of radiation effect.

(3) Shang *et al.* studied the effect of calorie restriction in B6C3F1 mice following exposure to 3.8 Gy of radiation at 1-week of age (Fig. 2) [26]. Because thymic lymphoma developed early in life, the survival curves declined rapidly during the first 200 days of life (Fig. 2A) which is also seen as a small peak in the mortality plot (Fig. 2B). Subsequently, after ~400 days of age, the mortality curve is high and above the control curve, or shifted toward younger ages, showing that the exclusion of thymic lymphoma does not lead to the disappearance of a radiation effect.

### Non-tumor deaths are affected or not by radiation exposures (page 162 of the Walburg paper)

This is the most important point of the argument. Walburg obtained the mouse autopsy data set reported by Upton *et al.* [27]. From the data set, Walburg selected individual data from female RFM mice acutely exposed to radiation and speculated on the cause of death from gross observation records which included tumor size etc. and found that no life-shortening was observed among individuals dying with non-tumor deaths. However, the number of mice in this category is only 29 in the 100 R group and 20 in the 300 R group (Table 2), which is too small to derive a firm conclusion.

**Table 2:** Adjusted mean age at death of RFM female mice exposed to 100 or 300 R of whole-body gamma radiation at 10 weeks of age versus controls^a^. (This table is a reproduction of Table 3 in the Walburg paper)

Cause of death	Treatment (R)	No. of animals	Adjusted mean age at death (days)^b^ ± SE	Difference from controls^c^
All causes	0	232	577 ± 8	
	100	95	552 ± 13	*P < 0.02*
	300	92	473 ± 17	*P < 0.01*
All causes	0	104	611 ± 10	
other than	100	29	650 ± 18	*P > 0.20*
neoplastic	300	20	657 ± 30	*P > 0.50*

This table is a copy from the Walburg paper to indicate that mean lifespan is not affected in mice which died from non-tumor deaths.

^a^From Upton *et al.* [27].

^b^Terminated at the age of 799 days.

^c^See Hoel and Walburg [28] for methods.

Walburg cited another example which used LAF1 male mice, and the results indicated that non-tumor deaths were not affected by irradiation at doses ~2 Gy (Fig. 3 is a reproduction of Fig. 4 in the Walburg paper). The cumulative mortality curve (adjusted) of the irradiated group was advanced by ~30 weeks for all causes of death (Fig. 3A**)** but overlapped with the control curve for non-cancer deaths (Fig. 3B). This result means that after adjustment of competing risks, non-cancer risks were not affected by the irradiation. Similar results are observed in females (Fig. 5 of the Walburg paper). However, the results are in sharp contrast to the results by Alexander who used the same LAF1 mice as mentioned earlier, that life shortening did occur in LAF1 mice bearing no tumors after exposure to 400 R (Fig. 3C) [1]. This discrepancy is unsolvable because the dataset used by Walburg is a part of the Upton’s 1960 study, and the number of mice and the new methods used to adjust for the competing risks were not presented.

**Fig. 3:**
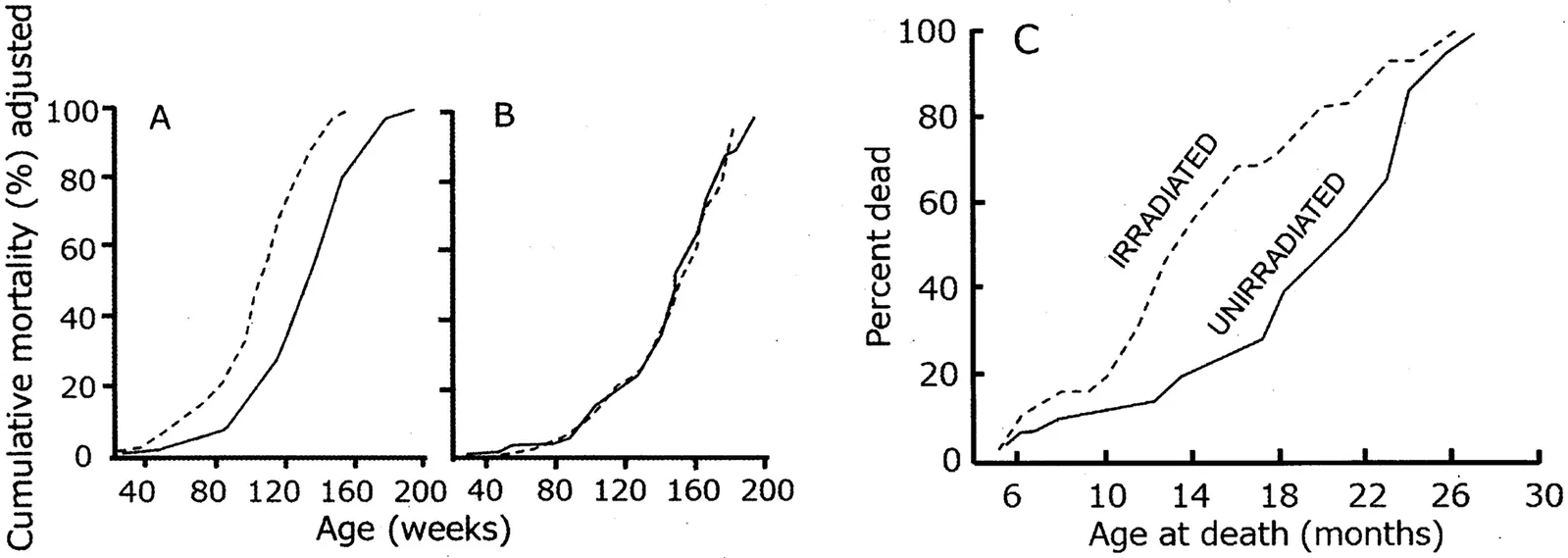
Adjusted cumulative mortality for LAF1 male mice exposed to ~2 Gy (179–265 rad). (A) All deaths, (B) non-tumor deaths. Solid lines represent controls, and broken lines represent irradiated groups (Fig. 4 of the Walburg paper [3]). (C) Life shortening in LAF1 mice irradiated with 400 R of X rays that died from non-tumor causes [1].

**Fig. 4:**
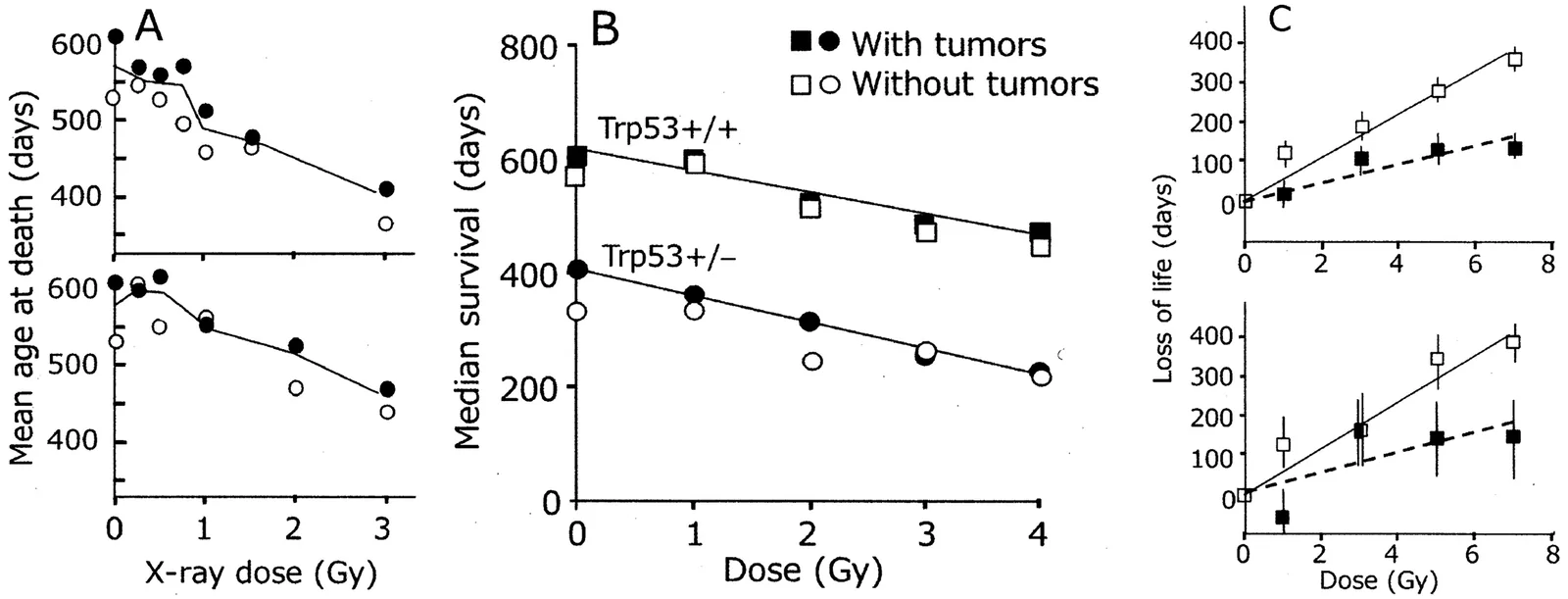
Radiation-induced life shortening in: (A) Tumor-bearing (●) and non-tumor bearing (○) male (top) and female (bottom) RF/Un mice [14]; (B) female B6.129S2 mice, wild or Trp53 heterozygotes [16] and (C) deaths from all causes (top) and non-tumor deaths (bottom panel) in male (■) and female (□) CBA/Cne mice [15].

**Fig. 5:**
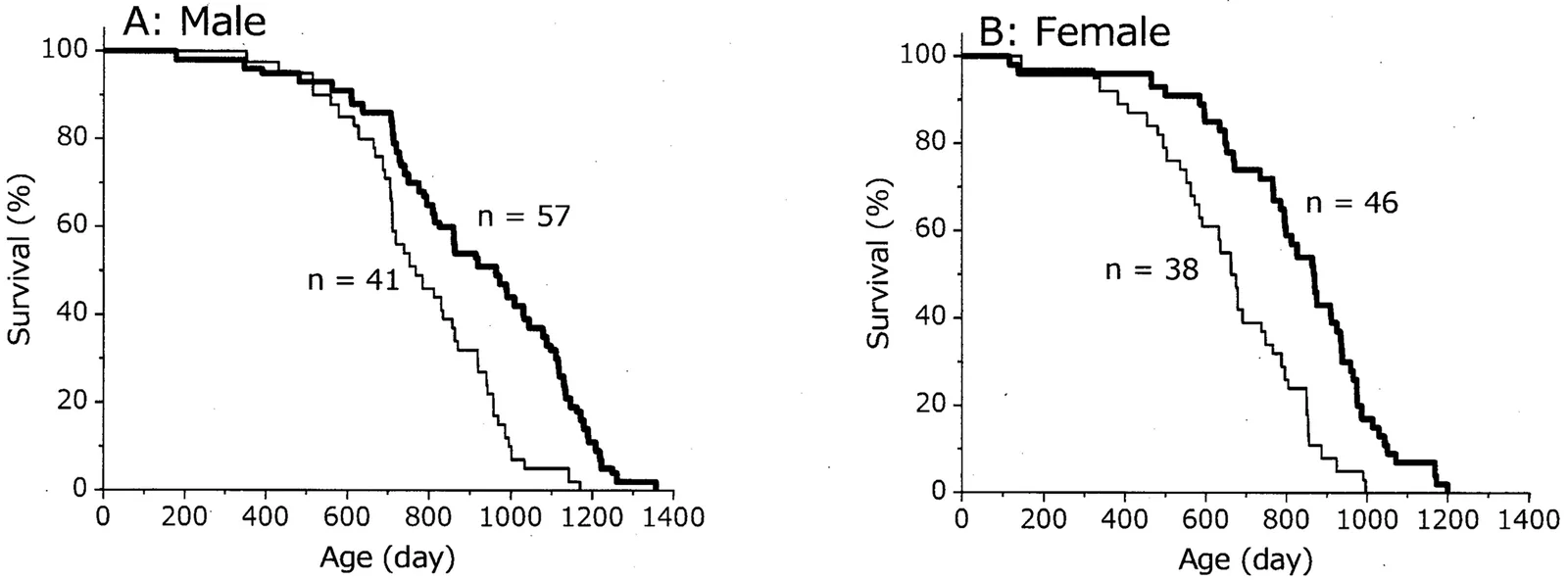
Radiation-induced life shortening in male (A) and female (B) B6C3F1 mice following long-term irradiation at low dose-rate (20 mGy/day) for 400 days (total 8 Gy) that died from non-tumor causes [8]. Thick and thin lines indicate unirradiated and irradiated mice, respectively. The numbers of mice are also indicated.

It is time now to present results of four additional studies which contradict Walburg’s notion. Fig. 4 shows that animals which died with and without tumors underwent similar life shortening. Fig. 4A represents the results by Upton *et al.* and shows that median age at death was equally reduced in both tumor-bearing (●) and non-tumor bearing deaths (○) [14]. The lines connect the mean ages of all deaths at each dose level and eventually pass close to the black circles (tumor-bearing deaths), indicating that the numbers of mice which died without tumors are relatively small. This Upton paper was published in 1967 in Radiation Research, 8 years prior to the Walburg paper, but was not mentioned in the Walburg review.

Fig. 4B represents results by Carlisle *et al.* and compared the mean lifespans of *Trp53* wild type and heterozygous mice (B6.129S2 females) in relation to the dose [16]. If one imagines that *Trp53* heterozygous animals are sensitive to the induction of radiation-related malignancies, one might expect that life-shortening effect of radiation could be larger in the heterozygotes (i.e. a steeper slope in Fig. 4B) but it was not the case as the slopes of the two curves were nearly equal. More importantly, mice dying with tumors and without tumors had a similar shortening of their lifespans.

Fig. 4C shows results by Di Majo *et al.* which used CBA/Cne mice of both sexes [15]. The upper panel shows the loss of lifespan for all animals, and the lower panel shows mice underwent non-tumor deaths to indicate that life shortening occurred equally in both tumor and non-tumor causes of death. Furthermore, radiation-induced life shortening was about twice as long in females (☐) than in males (■). In both instances, the sex-specific slopes were nearly equal between all causes and non-tumor deaths, suggesting that some common mechanisms are operating which are functionary different from mutagenesis model involved in the induction of malignancies.

The last example is a newly sorted data from the study by Tanaka *et al.* which was mentioned earlier [8]. While the study reported mainly tumors following a chronic irradiation of B6C3F1 mice (mean dose rate of 20 mGy/day × 400 days to give 8 Gy), slightly ˂10% of the mouse died from non-tumor causes. Fig. 5 shows survival curves of such non-tumor individuals (detailed pathologic data of individual mice are presented in the Supplementary file). It is clear that the lifespan is shortened in the irradiated group, and the mean lifespans were 914 days (male) and 822 days (female) for the unirradiated mice, and 785 days (male) and 656 days (female) for the irradiated mice. In other words, the mean lifespan was shortened by 129 days in males and 166 days in females by this type of exposure to radiation.

### Epidemiological studies in man (page 169 of the Walburg paper)

Walburg wrote here that epidemiologic studies of British radiologists [29] and atomic-bomb survivors [30] showed increased risks for cancers but not for non-cancers. However, we now know that since ~1990, non-cancer risks started to show significant increases among the survivors although the excess relative risk was small, about one fifth of that for cancer e.g. [31]. Thus, the lack of evidence in the 1970s does not mean that radiation does not affect non-cancer deaths in humans.

## DISCUSSION

It is unfortunate that radiation effects have long been thought to be relevant for tumor deaths, but not non-tumor deaths and this belief has lasted for ~50 years as a foundation in the field of radiation research. A possible reason for this seems most likely due to the small number of mice dying from non-tumor causes, which could give rise to unstable results; namely, either the lifespan was shortened or not in animals which died with non-tumor deaths. This was an inevitable consequence following selection of strains of mice for the experiments that were proven to respond well to radiation exposure, and the issue of non-tumor deaths was unlikely to be of primary interest. Also, we now know that some tumors (thymic lymphoma, acute myeloid leukemia and ovarian tumors) are easily induced in some strains of mice by radiation exposures while in most other fatal tumors, the proportion does not generally increase, which could be a source of confusion in understanding the late effects of radiation.

### Early onset model can better explain human epidemiological data

If we admit that life shortening occurs in mice dying tumor deaths and non-tumor deaths as well, then it becomes easier to understand several observations which remained unexplained by the traditional mutation induction model. Namely,

(1) In both humans and mice, solid cancer mortality curve shifts in parallel toward younger ages following an exposure to radiation [10, 13].

(2) The relative risk (RR) per Gy for cancer decreases with an increase in time since an exposure, and this occurs not only in atomic-bomb survivors, but also in mice. This is a natural consequence by the early onset model but is unexplained by the mutagenesis model [13].

(3) In the study of atomic-bomb survivors, RR values for cancer may look different among different organs, and it was recently found that the apparently different RR values come from difference in the shape of the background age-dependent mortality curve [32]. In this context, it is reminded that the RR values for cancer are usually expressed at an attained age of 70 because the value decreases with the increase of post-exposure time. Under such conditions, cancers with larger RR values (i.e. bladder, colon and lung) showed the baseline mortality curves which start to increase steeply at ~70 years of age, while those with smaller RR values (i.e. stomach, liver and esophagus) showed the baseline curves with a shallow increase or the incidence/mortality rate tended to level off after age 70.

### Why the non-cancer risk is low in humans

If we accept the notion that the life-shortening effect of radiation operates in both tumor and non-tumor deaths in mice, it is expected a priori that the same effect should also occur in humans. However, it is well understood that the RR for non-cancer death in humans is substantially smaller, about one fifth of the RR for cancers [31, 33–35]. Because baseline mortality curves for deaths other than cancer show a more rapid increase than that for cancer after age 70, it is expected that shifting the mortality curve toward younger ages would yield larger RR values for non-cancer deaths, but this is not the case. Therefore, one needs to speculate that in humans, the speed of radiation-induced accelerated aging may differ between organs which may lead to cancer or non-cancer deaths. To address the issue, we first need to know what the target cells are which may lead to non-cancer deaths; namely, heart and cerebrovascular diseases. Subsequently, measure cellular epigenetic clock such as developed by Horvath [36] to compare the speed of cellular aging in both species.

### Possible mechanisms of radiation-induced early onset of cancer and non-cancer diseases

Various models for radiation carcinogenesis were proposed in the past such as induction of mutation, genetic instability, tissue inflammation, reactive oxygen species, altered tissue microenvironment, etc. e.g. [37]. These events are rather acute effect, and none of those appears to play major roles either singly or in combination in radiation carcinogenesis because in mice and rats, radiation exposure at middle age or later cannot shorten the lifespan [38, 39]. This observation indicates that radiation-induced acute damage looks to be too crude to act additively on naturally occurring aging processes, and acute radiation damage requires a long latency period to become functional in causing accelerated aging.

Regarding the mechanisms of natural aging, different hypotheses are proposed such as accumulation of somatic mutations, error catastrophe, oxygen stress, failure of mitochondria function, alteration of protein structure and dedifferentiation of cells (epigenetic change) e.g. [40]. As mentioned above, as radiation does not induce life shortening in middle age or older animals, radiation-induced acute damage *per se* seems incapable of contributing to the life shortening, and it takes a long time to be transformed into a functional form(s). Thus, among various possibilities, the dedifferentiation model seems to fit most adequately the required conditions in radiation carcinogenesis. Namely, although radiation-induced DNA damage can be generally repaired correctly, it may not completely restore the three-dimensional chromatin structure (chromatin fatigue) because of altered methylation or re-localization of chromatin factors, which can lead to changes in the epigenetic timeclock e.g. [41]. In this regard, a recent in vivo study is worth mentioning here. Namely, Yang et al. created genetically engineered mice carrying an inducible restriction enzyme I-*Ppo*I. Induction of the gene by administration of Tamoxifen for 3 weeks led to advanced aging in terms of motor function, expression of inflammatory genes, cellular senescence etc. although the induced DNA breaks were all correctly repaired [42]. The results indicate that the carcinogenic action of radiation can be attributed to its production of DNA breaks as has been indicated for many years, which leads to accelerated organismal aging, and hence shifting the mortality rate toward younger ages. Here is one fundamental difference, however, from the traditional view; that is, mutation induction in tissue stem cells is not needed to consider. Table 3 shows the summary of the discussion.

**Table 3:** Possible mechanisms for radiation carcinogenesis vary depending on whether or not one accepts the existence of an effect on non-tumor deaths

	Mutagenesis model	Early onset model
Radiation effect	Only tumors	Tumor and non-tumor
Target	Tissue stem cells	Entire tissue
Action mechanism	Oncogenic mutation	Accelerated tissue aging
		via DNA damage repair

This table is the summary of Discussion.

## Supplementary Material

1_R_Supplementary_Data_rrag063

## PRESENTATION AT A CONFERENCE

The study is not presented at a conference.

## Clinical trial registration

No Clinical Trial Registration number.
