# Supplementary material for: Revisiting the Walburg hypothesis that radiation affects only tumor deaths but not non-tumor deaths in mice
Source: J Radiat Res. 2026 Sep 16;67(5):694–702. doi: 10.1093/jrr/rrag063 (PMC13650703; doi:10.1093/jrr/rrag063)
Supplement: Supplementary file 1 [file 1_r_supplementary_data_rrag063.pdf]

Supplementary Data. Individual lifespans of mice that died from non-neoplastic causes with or without non-lethal neoplasms

Animal ID	Lifespan days	Cause of death (COD)		Non Lethal Neoplasms (Organ of Origin)
**MALES (Non-Irradiated Control)**				
42	346	Respiratory System	Interstitial pneumonia	
149	481	Heart	Atrial thrombosis	Hepatocellular Adenoma (Liver)
156	609	Digestive System	Hemorrhagic colitis	
231	749	Respiratory System	Interstitial pneumonia	
287	561	Respiratory System	Interstitial pneumonia	Bronchiolo-Alveolar Carcinoma (Lung)
645	390	Respiratory System	Interstitial pneumonia, acute	Hepatocellular Carcinoma (Liver)
687	919	All Sites	Septicemia	Hepatocellular Adenoma (Liver)
708	740	Digestive System	Hemorragic enteritis of caecum, acute	
747	720	Respiratory System	Interstitial pneumonia, acute	Hepatocellular Adenoma (Liver)
939	727	Digestive System	Colitis, supprative, subacute, severe	Cortical Adenoma (Adrenal), Hepatocellular Adenoma (Liver), Follicular Cell Adenoma (Thyroid)
1024	972	All Sites	Panarteritis nodosa	Malignant Lymphoma (Hematopoietic), Hepatocellular Carcinoma (Liver)
1093	863	Heart	Myocarditis	Malignant Lymphoma (Hematopoietic)
1178	178	All Sites	Septicemia	
1606	1134	Heart	Heart failure	Hepatocellular Carcinoma (Liver)
1639	1116	Respiratory System	Interstitial pneumonia, acute	Hepatocellular Adenoma (Liver), Bronchiolo-alveolar Adenoma (Lung), Adenoma - Pars Intermedia (Pituitary), Fibrosarcoma (Soft Tissue)
1668	1162	Respiratory System	Interstitial pneumonia	Bronchiolo-alveolar Adenoma (Lung), Follicular Cell Adenoma (Thyroid)
1753	1222	All Sites	Septicemia	Bronchiolo-alveolar Adenoma (Lung)
1759	1132	Respiratory System	Interstitial pneumonia	Histiocytic Sarcoma (Hematopoietic)
1760	711	Heart	Pericarditis and pleuritis with secondary fibrosis	Histiocytic Sarcoma (Hematopoietic), Bronchiolo-alveolar Adenoma (Lung), Adenocarcinoma (Harderian Gland)
1975	775	Liver	Hepatic failure	
1993	1118	Respiratory System	Pulmonary congestive edema	Subcapsular Cell Adenoma (Adrenal), Malignant Lymphoma (Hematopoietic), Hepatocellular Adenoma (Liver), Renal Tubule Adenoma (Kidney)
2025	731	Respiratory System	Interstitial pneumonia	
2141	710	Respiratory System	Interstitial pneumonia, subacute	Choroid Plexus Papilloma (Brain)
2193	1191	Respiratory System	Interstitial pneumonia, subacute	Malignant Lymphoma (Hematopoietic), Bronchiolo-Alveolar Carcinoma (Lung), Follicular Cell Adenoma (Thyroid), Adenocarcinoma (Harderian Gland)
2262	787	Digestive System	Cecal Hemorrhage	Bronchiolo-alveolar Adenoma (Lung)
2278	914	All Sites	Septicemia	Bronchiolo-alveolar Adenoma (Lung)
2366	825	All Sites	Septicemia	Bronchiolo-alveolar Adenoma (Lung)
2413	1260	All Sites	Circulatory disturbance	Histiocytic Sarcoma (Hematopoietic), Bronchiolo-alveolar Adenoma (Lung)
2541	988	All Sites	Septicemia	
2612	610	Respiratory System	Pulmonary congestive edema	Adenoma (Coagulating Gland), Adenocarcinoma (Harderian Gland)
2695	637	Digestive System	Cecal Hemorrhage	
2763	535	Digestive System	Cecal Hemorrhage	Adenoma (Intestine)
2856	713	Respiratory System	Interstitial pneumonia, acute	
3091	809	Digestive System	Cecal Hemorrhage	Bronchiolo-Alveolar Carcinoma (Lung)
3193	1146	Respiratory System	Interstitial pneumonia	Cortical Adenoma (Adrenal), Bronchiolo-alveolar Adenoma (Lung)
3237	1032	Respiratory System	Interstitial pneumonia	Bronchiolo-Alveolar Carcinoma (Lung), Fibroma (Soft Tissue), Adenoma (Harderian Gland)
3263	794	Urinary System	Nephropathy	
3275	707	Respiratory System	Pulmonary congestive edema	
3292	1110	Heart	Myocarditis	Bronchiolo-alveolar Adenoma (Lung), Hemangiosarcoma (Vascular)
3465	860	Urinary System	Nephritis, supprative, severe	
3529	1129	Respiratory System	Interstitial pneumonia	Hemangioma (Vascular), Adenoma (Harderian Gland)
3584	1208	Heart	Myocardial necrosis	Hepatocellular Adenoma (Liver), Bronchiolo-alveolar Adenoma (Lung), Hemangiosarcoma (Vascular)
3602	814	Respiratory System	Interstitial pneumonia	Hepatocellular Adenoma (Liver)
3618	1249	Respiratory System	Interstitial pneumonia	Malignant Lymphoma (Hematopoietic)
3669	1189	Respiratory System	Interstitial pneumonia	Leydig Cell Adenoma (Testis), Malignant Lymphoma (Hematopoietic), Bronchiolo-alveolar Adenoma (Lung), Leiomyosarcoma (Soft Tissue)
3737	861	Respiratory System	Interstitial pneumonia, supprative	Hepatocellular Carcinoma (Liver)
3747	1357	Respiratory System	Interstitial pneumonia	Bronchiolo-alveolar Adenoma (Lung), Adenoma (Harderian Gland)
3827	1030	Respiratory System	Pulmonary congestive edema	Malignant Lymphoma (Hematopoietic), Hepatocellular Adenoma (Liver)
3831	1087	Respiratory System	Interstitial pneumonia	Malignant Lymphoma (Hematopoietic), Hepatocellular Adenoma (Liver), Bronchiolo-alveolar Adenoma (Lung)
3856	1044	Respiratory System	Interstitial pneumonia	Bronchiolo-alveolar Adenoma (Lung)
3864	1078	Other	Peritonitis	
3913	990	Respiratory System	Interstitial pneumonia	Malignant Lymphoma (Hematopoietic)
3920	1097	Respiratory System	Interstitial pneumonia	Hepatocellular Adenoma (Liver)
3923	1008	Respiratory System	Interstitial pneumonia	Malignant Lymphoma (Hematopoietic), Fibroma (Soft Tissue)
3959	1179	All Sites	Septicemia	Hepatocellular Carcinoma (Liver)
3966	1171	Respiratory System	Interstitial pneumonia	Adenoma (Intestine), Hepatocellular Adenoma (Liver)
3982	1218	Respiratory System	Interstitial pneumonia	Hepatocellular Carcinoma (Liver)
**MALES (20 mGy/day x 400 d = 8 Gy)**				
41	431	Respiratory System	Interstitial pneumonia, severe	
72	514	Respiratory System	Interstitial pneumonia, subacute	Bronchiolo-Alveolar Adenoma (Lung), Adenoma (Harderian Gland)
139	687	Other	Peritonitis	Hepatocellular Adenoma (Liver), Bronchiolo-Alveolar Carcinoma (Lung)
145	692	Heart	Atrial thrombosis	Adenoma, Leydig Cell (Testis), Follicular Cell Adenoma (Thyroid)
217	739	Urinary System	Hyaline Glomerulopathy	

Animal ID	Lifespan days	Cause of death (COD)		Non Lethal Neoplasms (Organ of Origin)
239	752	Respiratory System	Pneumonia, Acidophilic Macrophage	Hepatocellular Adenoma (Liver)
487	664	Heart	Valvular endocarditis	Malignant Lymphoma (Hematopoietic), Hemangioma (Vascular)
703	857	Digestive System	Enteritis	
804	919	Urinary System	Nephropathy	Hepatocellular Adenoma (Liver), Bronchiolo-Alveolar Adenoma (Lung), Hemangiosarcoma (Vascular)
838	707	Heart	Heart failure	Osteosarcoma, Adenoma (Harderian Gland)
895	784	Hematopoietic System	Myelophthisis	Adrenal gland Cortical Adenoma, Hepatocellular Adenoma (Liver), Adenoma (Harderian Gland)
896	719	All Sites	Panarteritis nodosa	Bronchiolo-Alveolar Adenoma (Lung), Adenoma (Harderian Gland)
1108	958	Pancreas	Subacute pancreatitis	Bronchiolo-Alveolar Adenoma (Lung), Bronchiolo-Alveolar Carcinoma (Lung), Adenoma (Harderian Gland)
1219	986	Respiratory System	Interstitial pneumonia	Adenoma (Intestine), Bronchiolo-Alveolar Carcinoma (Lung), Adenoma (Harderian Gland)
1279	969	All Sites	Septicemia	
1502	943	Digestive System	Ulcerative colitis (with 2ア peritonitis)	Hepatocellular Carcinoma (Liver), Follicular Cell Adenoma (Thyroid), Adenocarcinoma (Harderian Gland)
1660	578	Respiratory System	Interstitial pneumonia, acute	Rhabdomyosarcoma
1781	627	Respiratory System	Pulmonary congestive edema	
1877	767	Heart	Cardio-pulmonary failure	Medullary Tumor - Benign (Adrenal), Hepatocellular Adenoma (Liver), Choroid Plexus Papilloma (Brain), Hemangioma (Vascular)
1878	705	Heart	Cardio-pulmonary failure	Bronchiolo-Alveolar Carcinoma (Lung), Choroid Plexus Papilloma (Brain)
1969	711	Liver	Hepatic failure	Adenocarcinoma (Intestine), Bronchiolo-Alveolar Carcinoma (Lung)
1991	812	Heart	Cardiomyopathy	Bronchiolo-Alveolar Adenoma (Lung), Harderian Gland Adenocarcinoma
2137	711	Soft Tissue	Myositis	Hepatocellular Adenoma (Liver), Bronchiolo-Alveolar Adenoma (Lung)
2154	352	Urinary System	Pyelonephritis, acute	Hepatocellular Adenoma (Liver)
2207	863	All Sites	Septicemia	Bronchiolo-Alveolar Adenoma (Lung), Choroid Plexus Papilloma (Brain)
2221	830	Respiratory System	Pulmonary congestive edema	Myoepithelioma (Salivary Gland), Hepatocellular Adenoma (Liver)
2338	558	All Sites	Septicemia	Bronchiolo-Alveolar Adenoma (Lung)
2422	615	Respiratory System	Interstitial pneumonia, chronic	Hepatocellular Adenoma (Liver)
2693	515	Urinary System	Nephropathy	
2711	668	Other	Pleuritis	Hepatocellular Adenoma (Liver)
2761	710	Respiratory System	Interstitial pneumonia, subacute	Hepatocellular Adenoma (Liver), Bronchiolo-Alveolar Adenoma (Lung), Adenoma (Harderian Gland)
2990	1001	Respiratory System	Interstitial pneumonia, subacute	Adenoma (Intestine), Bronchiolo-Alveolar Carcinoma (Lung), Keratoacanthoma (Skin)
3573	1142	Respiratory System	Interstitial pneumonia	Adenoma (Harderian Gland)
3582	957	Heart	Endo-myocarditis, chronic, supprative	Hepatocellular Carcinoma (Liver), Bronchiolo-Alveolar Carcinoma (Lung), Adenoma (Harderian Gland)
3617	940	Respiratory System	Pulmonary congestive edema	Malignant Lymphoma (Hematopoietic), Hepatocellular Adenoma (Liver), Adenoma (Harderian Gland)
3633	920	Respiratory System	Interstitial pneumonia	Adrenal Gland Benign Medullary Tumor, Hepatocellular Adenoma (Liver)
3636	1170	Respiratory System	Interstitial pneumonia	Malignant Lymphoma (Hematopoietic), Bronchiolo-Alveolar Adenoma (Lung), Adenoma (Harderian Gland)
3645	834	Respiratory System	Interstitial pneumonia	
3755	870	Respiratory System	Interstitial pneumonia	Hepatocellular Adenoma (Liver), Bronchiolo-Alveolar Adenoma (Lung)
3771	995	Respiratory System	Pulmonary congestive edema	Islet Cell Adenoma (Pancreas), Hepatocellular Adenoma (Liver), Bronchiolo-Alveolar Adenoma (Lung)
3943	1033	Respiratory System	Interstitial pneumonia	Hemangiosarcoma (Vascular)
**FEMALES (Non-Irradiated Control)**				
3	137	Urinary System	Hyaline glomerulopathy	
9	116	Respiratory System	Interstitial pneumonia	
60	598	Respiratory System	Pulmonary congestive edema	
127	648	Heart	Tamponade	
332	669	Respiratory System	Interstitial pneumonia, acute	Bronchiolo-Alveolar Carcinoma (Lung)
391	786	Reproductive System	Hemorrhage from paraovarian cyst	
509	735	All Sites	Septicemia	
527	799	Urinary System	Nephropathy	Adenoma - Pars Distalis (Pituitary) , Hemangioma (Vascular)
585	671	Respiratory System	Interstitial pneumonia	
716	924	All Sites	Septicemia	Cortical Adenoma (Adrenal), Hepatocellular Adenoma (Liver), Follicular Cell Adenoma (Thyroid)
753	813	Digestive System	Cecal Hemorrhage	Bronchiolo-alveolar Adenoma (Lung)
862	933	Pancreas	Cyst, Pancreas	Malignant Lymphoma (Hematopoietic)
936	766	Reproductive System	Internal hemorrhage due to ruptured Hemorrhagic cy	Malignant Lymphoma (Hematopoietic)
940	795	Digestive System	Colitis, supprative, subacute, severe	Hepatocellular Carcinoma (Liver)
1089	500	Urinary System	Nephropathy	
1130	1029	Heart	Valvular endocardiosis	Bronchiolo-alveolar Adenoma (Lung), Follicular Cell Adenoma (Thyroid)
1318	1043	Other	Peritonitis	Hepatocellular Adenoma (Liver), Bronchiolo-Alveolar Carcinoma (Lung)
1325	465	All Sites	Amyloidosis	
1330	1014	Digestive System	Supprative enteritis (ileum)	Adenoma - Pars Distalis (Pituitary) , Follicular Cell Adenoma (Thyroid)
1426	585	Urinary System	Hyaline Glomerulopathy	
1643	635	Digestive System	Cecal Hemorrhage	Adenoma (Harderian Gland)
1893	1168	Respiratory System	Interstitial pneumonia, subacute	Luteoma - Benign (Ovary)
1994	1172	Heart	Cardiomyopathy	Adenoma - Pars Distalis (Pituitary), Follicular Cell Adenoma (Thyroid)
2044	768	Reproductive System	Pyometra	
2073	1200	Respiratory System	Interstitial pneumonia, acute	Hepatocellular Carcinoma (Liver), Hemangioma (Vascular)
2190	796	Respiratory System	Interstitial pneumonia, chronic	Cystadenoma (Ovary)
2203	827	Urinary System	Renal amyloidosis	

Animal ID	Lifespan days		Cause of death (COD)	Non Lethal Neoplasms (Organ of Origin)
2224	867	Urinary System	Renal amyloidosis	
2225	867	Respiratory System	Interstitial pneumonia	Endometrial Stromal Polyp (Uterus), Malignant Schwannoma (Soft Tissue)
2451	875	Reproductive System	Pyometra	Fibroma (Vagina), Hepatocellular Adenoma (Liver), Malignant Schwannoma (Soft Tissue)
2582	974	All Sites	Septicemia	
2653	958	Respiratory System	Interstitial pneumonia, chronic	
2665	870	Liver	Hepatic failure	
2754	652	Urinary System	Hyaline glomerulopathy	
2797	974	Respiratory System	Interstitial pneumonia, chronic	Leiomyoma (Uterus)
2907	975	Respiratory System	Pulmonary fibrosis	
2968	596	Urinary System	Hyaline glomerulopathy	
3448	908	Respiratory System	P.Carinii pneumonia	
3604	935	Respiratory System	Interstitial pneumonia, chronic	Hepatocellular Adenoma (Liver)
3616	911	Other	Pleuritis	
3692	864	Respiratory System	Interstitial pneumonia	Malignant Lymphoma (Hematopoietic), Bronchiolo-alveolar Adenoma (Lung)
3816	1050	Liver	Hepatitis	
3875	965	Respiratory System	Interstitial pneumonia	Cystadenoma (Ovary)
3876	937	Respiratory System	Interstitial pneumonia	Malignant Lymphoma (Hematopoietic)
3892	1071	Respiratory System	P.Carinii pneumonia	Cystadenoma (Ovary)
3903	986	Respiratory System	P.Carinii pneumonia	
**FEMALES (20 mGy/day x 400 d = 8 Gy)**				
1	145	Urinary System	Nephropathy	
14	324	All Sites	Septicemia	Bronchiolo-Alveolar Adenoma (Lung)
35	383	Urinary System	Hyaline glomerulopathy	
40	338	Respiratory System	Interstitial pneumonia	
57	591	Respiratory System	Interstitial pneumonia	
112	563	Respiratory System	Interstitial pneumonia	Granulosa Cell Tumor - Benign (Ovary), Hemangiosarcoma (Vascular)
129	495	All Sites	Septicemia	Bronchiolo-Alveolar Adenoma (Lung)
143	537	Urinary System	Nephropathy	Malignant Lymphoma (Hematopoietic), Bronchiolo-Alveolar Adenoma (Lung)
164	679	Respiratory System	Interstitial pneumonia (subacute)	
184	691	Respiratory System	Alveolar collapse (atelectasis)	Subcapsular Cell Adenoma (Adrenal)
223	679	Urinary System	Hyaline Glomerulopathy	Subcapsular Cell Adenoma (Adrenal), Malignant Lymphoma (Hematopoietic), Hepatocellular Adenoma (Liver)
278	584	Urinary System	Hyaline Glomerulopathy	
398	662	All Sites	Septicemia	Subcapsular Cell Adenoma (Adrenal), Adenoma (Harderian Gland)
490	850	Heart	Heart failure (due to multiple tumors)	Tubulostromal Adenocarcinoma (Ovary), Hepatocellular Adenoma (Liver), Bronchiolo-Alveolar Adenoma (Lung), Hemangiosarcoma, Adenoma (Harderian Gland)
591	886	Respiratory System	Interstitial pneumonia	Bronchiolo-Alveolar Adenoma (Lung)
592	766	Respiratory System	Pneumonia and Pleuritis	Tubulostromal Adenoma (Ovary), Hepatocellular Adenoma (Liver)
729	804	Respiratory System	Interstitial pneumonia	Subcapsular Cell Adenoma (Adrenal)
812	856	Respiratory System	Pneumonia, Interstitial	Adenoma - Pars Distalis (Pituitary), Hemangioma (Vascular)
971	408	Respiratory System	Interstitial pneumonia	Adenoma (Harderian Gland)
1053	796	All Sites	Septicemia	
1167	455	Urinary System	Hyaline glomerulopathy	
1393	573	Respiratory System	Interstitial pneumonia	Adenoma (Harderian Gland)
1731	633	Pancreas	Pancreatitis, Chronic	Medullary Tumor - Benign (Adrenal)
1803	996	Respiratory System	Interstitial pneumonia, chronic	Cystadenoma (Ovary), Tubulostromal Adenoma (Ovary), Bronchiolo-Alveolar Adenoma (Lung), Adenoma (Harderian Gland)
1898	748	Urinary System	Renal Amyloidosis	Tubulostromal Adenoma (Ovary), Malignant Lymphoma (Hematopoietic), Rhabdomyosarcoma (Soft Tissue)
1921	663	Nervous System	Hemorrhage into the brain and spinal cord	Bronchiolo-Alveolar Carcinoma (Lung)
2253	503	Respiratory System	Interstitial pneumonia, chronic	Subcapsular Cell Adenoma (Adrenal)
2291	853	Respiratory System	Interstitial pneumonia, subacute	Tubulostromal Adenoma (Ovary), Squamous Cell Papilloma (Skin)
2303	854	Respiratory System	Interstitial pneumonia, chronic	Subcapsular Cell Adenoma (Adrenal), Cystadenoma (Ovary), Malignant Lymphoma (Hematopoietic), Bronchiolo-Alveolar Adenoma (Lung)
2489	481	Reproductive System	Internal hemorrhage due to ruptured Hemorrhagic cy	
2546	923	Respiratory System	Interstitial pneumonia, chronic	Malignant Lymphoma (Hematopoietic)
2728	552	Reproductive System	Internal hemorrhage due to ruptured Hemorrhagic cy	
2845	990	Respiratory System	Interstitial pneumonia, chronic	Tubulostromal Adenoma (Ovary)
3016	637	Urinary System	Hyaline glomerulopathy	Rhabdomyosarcoma (Soft Tissue), Fibrosarcoma (Soft Tissue)
3046	738	Other	Peritonitis	
3156	675	Respiratory System	Pulmonary congestive edema	Islet Cell Adenoma (Pancreas)
3243	787	Respiratory System	Interstitial pneumonia, chronic	Tubulostromal Adenoma (Ovary), Malignant Lymphoma (Hematopoietic)
3671	849	Respiratory System	Interstitial pneumonia, chronic	Tubulostromal Adenoma (Ovary), Bronchiolo-Alveolar Carcinoma (Lung) , Adenoma (Mammary gland), Fibroma (Soft Tissue)
